# Clinical Implications of Hedgehog Pathway Signaling in Prostate Cancer

**DOI:** 10.3390/cancers7040871

**Published:** 2015-09-29

**Authors:** Daniel L. Suzman, Emmanuel S. Antonarakis

**Affiliations:** Johns Hopkins Sidney Kimmel Comprehensive Cancer Center, 1650 Orleans Street, CRB1-1M45, Baltimore, MD 21287, USA

**Keywords:** Hedgehog pathway, prostate cancer

## Abstract

Activity in the Hedgehog pathway, which regulates GLI-mediated transcription, is important in organogenesis and stem cell regulation in self-renewing organs, but is pathologically elevated in many human malignancies. Mutations leading to constitutive activation of the pathway have been implicated in medulloblastoma and basal cell carcinoma, and inhibition of the pathway has demonstrated clinical responses leading to the approval of the Smoothened inhibitor, vismodegib, for the treatment of advanced basal cell carcinoma. Aberrant Hedgehog pathway signaling has also been noted in prostate cancer with evidence suggesting that it may render prostate epithelial cells tumorigenic, drive the epithelial-to-mesenchymal transition, and contribute towards the development of castration-resistance through autocrine and paracrine signaling within the tumor microenvironment and cross-talk with the androgen pathway. In addition, there are emerging clinical data suggesting that inhibition of the Hedgehog pathway may be effective in the treatment of recurrent and metastatic prostate cancer. Here we will review these data and highlight areas of active clinical research as they relate to Hedgehog pathway inhibition in prostate cancer.

## 1. Introduction

Prostate cancer is the second most common cause of cancer-related deaths in the United States, killing approximately 30,000 men per year [1]. Despite advances in the detection and treatment of prostate cancer using optimal local and systemic therapies including surgery, radiation therapy, and androgen deprivation therapy, many prostate cancers will persist after primary therapy and eventually become refractory to hormonal and cytotoxic therapies. While additional therapies have been approved that extend survival in castration-resistance prostate cancer, none of these are curative. Novel drugs, particularly those that may impact the tumor microenvironment and reverse resistance to hormonal and cytotoxic therapies, are thus needed.

The Hedgehog (Hh) signaling pathway is an evolutionarily-conserved developmental pathway that may be a useful target for cancer therapies. Hh signaling regulates epithelial and mesenchymal interactions in a variety of tissues during mammalian embryogenesis [2]. The extracellular Hh ligands (Sonic Hh (SHH), Indian Hh (IHH), or Desert Hh (DHH)) bind to Patched (PTCH1), a cell-surface transmembrane protein receptor, which relieves the inhibitory effect of PTCH1 on Smoothened (SMO), a transmembrane protein belonging to the G protein-coupled receptor superfamily [3]. Signal transduction by SMO then leads to activation and nuclear localization of transcription factors (e.g., GLI1) regulating expression of target Hh genes, which control cell proliferation, survival, differentiation, and angiogenesis [2].

In adults, the Hh pathway maintains homeostasis of several human organs and tissues including the stem cell populations of the skin and central nervous system, but is otherwise largely quiescent. Genetic alterations in the Hh pathway leading to constitutive activation of Hh signaling, such as inactivating mutations of *PTCH1* or gain-of-function mutations in *SMO*, are linked to the development of several human malignancies such as basal cell carcinoma (BCC), medulloblastoma, and rhabdomyosarcoma [4,5]. However, aberrant Hh signaling without evidence of germline or somatic genetic defects in Hh pathway proteins has also been associated with other tumors including prostate cancer.

## 2. The Role of the Hedgehog Pathway in Prostate Cancer

Normal prostate development involves Hh pathway activation in the epithelial and stromal compartments leading to ductal morphogenesis via concentrated SHH expression at sites of ductal bud formation. In the adult prostate gland, the pathway is involved in regenerating prostate epithelial cells and regulating their differentiated state [6]. Over-expression of Hh signaling, however, has been noted in prostate cancer in both animal models and human samples. Early reports exploring the role of inhibiting the pathway via cyclopamine or anti-SHH antibodies suggested a direct role for the Hh pathway in the development and progression of prostate cancer [7]. In this context, Hh signaling may promote tumor formation from prostatic epithelial cells, drive the epithelial-to-mesenchymal transition (EMT) which is associated with tumor invasion, and contribute towards the development of castration-resistance and chemotherapy-resistance.

The pathway appears complex and may reflect interactions within the tumor microenvironment between tumor cells and stromal cells. Within the microenvironment, the localization of expression of the Hh pathway and the nature of the interaction between epithelium and stromal components remains an area of controversy. For example, in one study of xenografted tumors, *GLI1* mRNA localized to the stromal compartment while SHH localized to the prostatic epithelium, indicating active paracrine Hh signaling from the tumor in the surrounding stroma. [8] However, in a study evaluating human prostate tissue, *in situ* hybridization of GLI1 mRNA localized to the epithelium but not to the surrounding stroma and was co-expressed with PTCH1 and SHH, suggesting autocrine Hh signaling [8,9]. Tzelepi *et al.* found that epithelial expression of GLI1, SHH, SMO, and PTCH by immunohistochemistry was higher in primary prostate carcinomas compared with non-neoplastic peripheral zone tissue, but was lower in the surrounding stromal tissue. Higher-grade and higher-stage prostate cancers demonstrated even lower stromal localization of PTCH, with the lowest expression occurring in metastatic bone lesions [10]. Thus, the Hh pathway components appear to be differentially expressed in the tumor microenvironment as compared to benign tissues. The issue of whether clinically relevant Hh signaling in prostate cancer occurs via an autocrine or paracrine model remains an open question.

The Hh pathway may be particularly active in men with hormone-naïve localized prostate cancer at high risk for metastatic spread compared with low-risk tumors. Gene expression profiles from localized high-grade prostate tumors differed in men who either rapidly developed metastases within the first 5 years following radical prostatectomy *versus* those men who were metastasis-free for >5 years after surgery. In men who developed early metastases, embryonic stem cell pathways, including the Hh and Notch pathways, were highly differentially expressed compared with the metastasis-free group as determined by gene expression profiling, and *SHH* was up-regulated 3.7-fold in the early-metastasis cohort, suggesting increased Hh signaling in localized prostate cancer with metastatic potential [11]. Similarly, Kim *et al.* evaluated 155 radical prostatectomy specimens from men with localized prostate cancers via immunohistochemistry and found increased expression of multiple components of the Hh pathway, including SHH, PTCH1, SMO, and GLI. In a multivariate model, increased SHH expression was an independent prognostic factor for biochemical recurrence beyond clinical factors that included Gleason score, stage, tumor volume, and pretreatment PSA [12].

**Figure 1:**
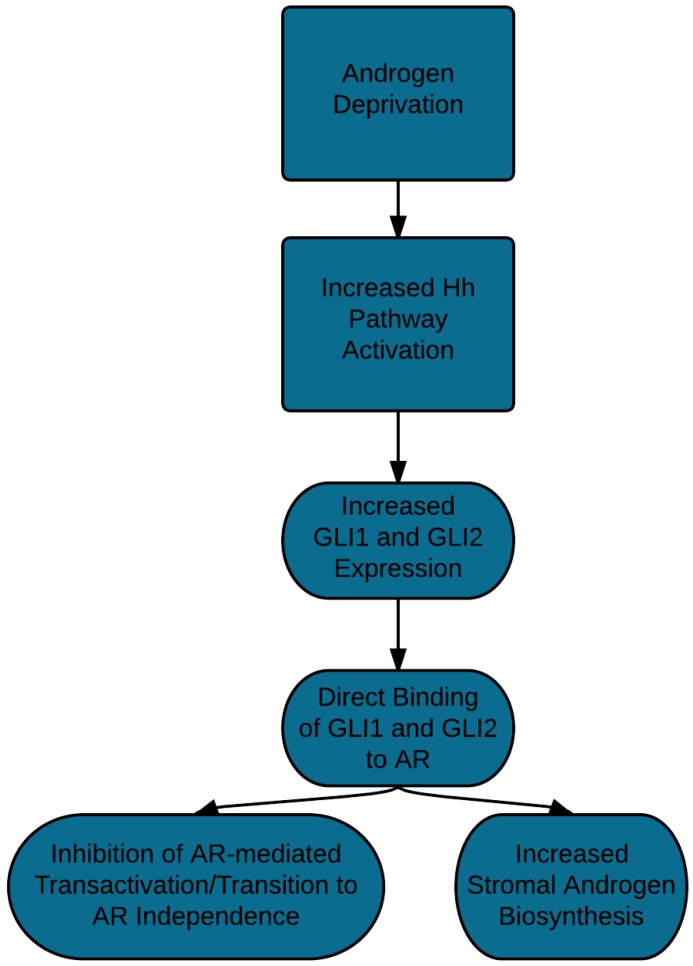
Putative mechanisms of crosstalk between the androgen receptor (AR) and Hh pathways.

Cross-talk between the Hh and androgen signaling pathways has been noted both *in vitro* and in human radical prostatectomy specimens (Figure 1). For example, administration of dihydrotestosterone (DHT) to pregnant mice with *Shh*-mutant fetuses was sufficient to rescue prostatic budding [13]. Additionally, in a low-androgen environment, both cyclopamine and siRNA directed against *SMO* caused downregulation of androgen-regulated genes in prostate cancer cells while administration of exogenous GLI1 allowed cell growth in an androgen-deficient medium [14]. In addition, Hh signaling may promote the development of castration resistance through induction of steroidogenic activity in prostate cancer cells via paracrine signaling. For example, Levina *et al.* demonstrated increased gene expression of cholesterol/steroid biosynthetic pathways following administration of a Hh agonist and further demonstrated the subsequent increased output of testosterone from the adrenal precursor: dihydroepiandrosterone (DHEA) [15]. Similarly, Sirab *et al.* demonstrated the mutual interaction between the androgen receptor (AR) and Hh pathways. Dihydrotestosterone (DHT) administration inhibits SHH in prostate cancer cell lines while administration of cyclopamine modulates the activity of the androgen receptor and can attenuate cell proliferation and AR signaling induced by dihydrotestosterone [16]. This interaction may occur at the level of GLI1 and GLI2 given that co-immunoprecipitation experiments have demonstrated that these transcription factors can bind directly to the androgen receptor protein [17].

The correlation between advanced disease state and hormonal resistance with Hh pathway expression provides additional evidence of an interaction between the two pathways. For example, malignant prostate tissue evaluated retrospectively from radical prostatectomy specimens demonstrated increased levels of GLI1 protein (using immunohistochemical staining) compared to benign prostatic epithelium; elevated GLI1 levels were also correlated with increasing tumor grade. Higher Hh signaling expression also correlated with increased tumor size, higher pre-treatment PSA levels, and more advanced stage [12]. Azoulay *et al.* assessed specimens from a wide range of prostate cancer disease states and found that epithelial expression of SHH became elevated following hormonal therapy compared with the hormone-naïve state [18]. In another study, circulating tumor cells (CTCs) from men with metastatic castration-resistant prostate cancer demonstrated significantly higher PTCH expression compared with prostate samples from normal individuals, with a positive correlation between higher PTCH expression and longer duration of androgen-targeted therapy [19].

Inhibiting the Hh pathway through small-molecule SMO inhibitors appears fruitful in pre-clinical models of castration-resistant prostate cancer (CRPC). For example, the potent SMO inhibitor TAK-441, demonstrated decreased tumor growth in CRPC xenograft models. These responses were associated with decreased GLI1 expression and decreased Ki67 staining [20]. Additionally, treatment with the SMO inhibitor, vismodegib, reduced the proliferation rate of a xenograft derived from the bone metastasis of a patient with CRPC, although no difference in tumor volume was noted. Of interest, treatment with vismodegib was associated with inhibition of the Hh pathway in the stroma, but not epithelium, in that study [21]. Finally, the combination of Hh inhibition via sonidegib or vismodegib with androgen receptor signaling inhibition via pyrvinium pamoate was synergistic in inhibiting the growth of CRPC cells and xenografts [22].

Chemotherapy may further upregulate the Hh signaling pathway and promote eventual chemotherapy resistance. In a retrospective analysis of 53 patients treated pre-operatively with androgen ablation with or without chemotherapy (KAVE (ketoconazole, doxorubicin, vinblastine, and estramustine)) compared to stage-matched and grade-matched controls, Efstathiou *et al.* found that whereas untreated specimens demonstrated limited and heterogeneous GLI2 expression in both epithelial and stromal compartments, treatment with androgen ablation (and particularly with androgen ablation plus chemotherapy) was associated with increased and homogeneous expression and nuclear localization of GLI1 and GLI2. Notably, SHH ligand expression in the stroma was strongly associated with expression of downstream components of the Hh pathway, lending further support to the notion of paracrine Hh signaling in this setting [23].

Additionally, Domingo-Domenech *et al.* determined that docetaxel-resistant prostate cancer cells over-expressed the Hh and Notch pathways. Inhibition of either pathway suppressed tumor growth while inhibition of both demonstrated synergistic effects. Downstream interaction with the AKT and Bcl-2 pathways was thought to mediate this synergy [24]. Aberrant Hh pathway activity may also promote “stemness” of prostate cancer cells—a putative phenotype resistant to androgen deprivation therapy, chemotherapy, and radiation. In support of this notion, Nanta *et al.* found that the SMO inhibitor, sonidegib, suppressed the epithelial-to-mesenchymal transition of the putative prostate cancer stem-cell phenotype CD133+/CD44+ cells and reduced their sphere-forming ability, while increasing apoptosis [25]. In another study, treatment with vismodegib in combination with docetaxel enhanced apoptosis in a stem-like population of PC3 prostate cancer cells expressing high levels of multidrug resistant proteins and synergistically inhibited a PC3 xenograft model *in vivo* [26]. There are currently no published data evaluating the combination of chemotherapy and Hh inhibition in patients with prostate cancer; however, a pilot trial of vismodegib in combination with gemcitabine in metastatic pancreatic cancer did not demonstrate superior clinical responses compared with gemcitabine alone despite achieving intratumoral Hh downmodulation [27].

## 3. Clinical Experience with Hedgehog Inhibitors in Prostate Cancer

Despite promising preclinical results, there have been limited clinical data generated to date using Hh pathway inhibitors in prostate cancer patients. The early data, however, appear promising and suggest that further clinical investigation warranted.

**Figure 2:**
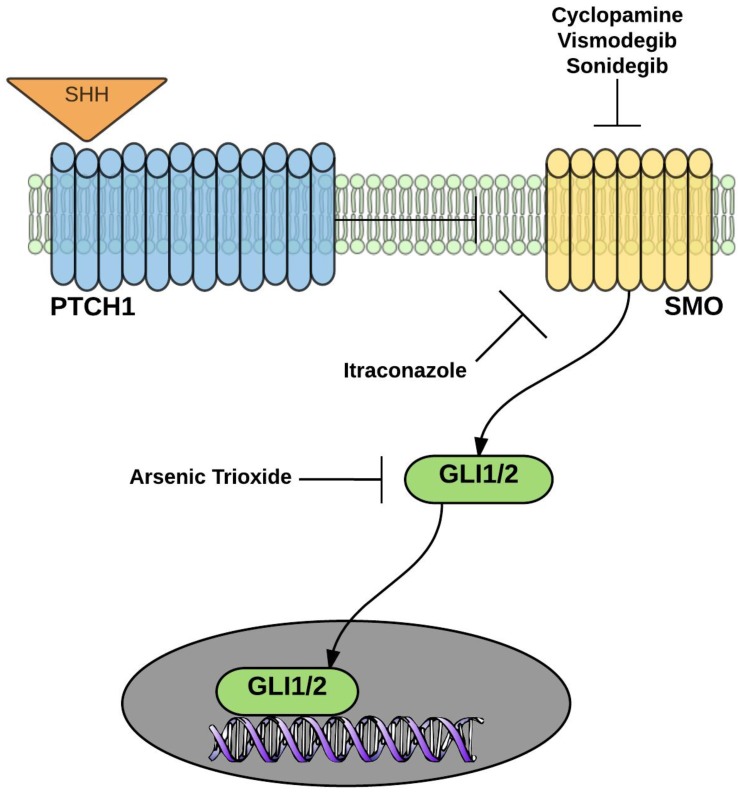
Binding of the Hedgehog ligand (SHH) to Patched (PTCH1) removes the inhibitory signal for the G-protein-coupled receptor, Smoothened (SMO), causing translocation of the GLI1 and GLI2 transcription factors into the nucleus. Itraconazole inhibits SMO at a site distinct from cyclopamine, vismodegib, and sonidegib. Arsenic trioxide degrades GLI2 and, in combination with itraconazole, demonstrates activity in animal models resistant to vismodegib due to SMO mutations.

The antifungal drug, itraconazole, was identified as a putative Hh pathway inhibitor via a GLI-luciferase reporter screen, a property unique to itraconazole and not shared by other azole antifungals [28]. Mechanistically, itraconazole acts as a SMO inhibitor at a site distinct from other SMO antagonists (including vismodegib, sonidegib, and cyclopamine,) though with 100-fold lower potency *in vitro* and with 65% pathway inhibition after one month of therapy in contrast to 90% pathway inhibition with vismodegib (Figure 2) [28,29]. As with vismodegib, itraconazole has demonstrated clinical activity in patients with recurrent or metastatic BCC [30]. Interestingly, in patients with advanced BCC, itraconazole often retained efficacy even in those with acquired resistance to vismodegib, further speaking to a non-overlapping mechanism of action.

The bulk of the clinical experience with itraconazole in prostate cancer derives from a single randomized phase II study conducted in men with metastatic castration-resistant prostate cancer (CRPC) [31]. This study targeted patients with resistance to conventional hormonal therapies, and was largely performed in the pre-abiraterone and pre-enzalutamide era; none of the patients had received prior cytotoxic chemotherapy. Men were randomized to receive either itraconazole given at a high dose (600 mg/day) or at a low dose (200 mg/day), with the primary endpoint of PSA progression-free survival (PPFS) after 24 weeks of therapy. While the low-dose arm closed early due to a pre-specified futility assessment, the high-dose arm was associated with clinical activity yielding a delayed PSA progression of 48% at 24 weeks with median PPFS of 17 weeks. In the high-dose arm, the 24-week radiographic progression-free survival (PFS) rate was 61.6% and the median PFS was 35.9 weeks, which compared favorably with PFS estimates of other historical FDA-approved agents. Notably, 14.3% of patients experienced a PSA response (≥50% decline in PSA). In addition, some patients demonstrated tumor shrinkages in soft-tissue metastases (11.1% partial objective response). Common side effects of itraconazole (especially at the high dose: 600 mg/day) included rash, fatigue, nausea, constipation, and peripheral edema with some grade-3 toxicities such as hypokalemia and hypertension. Interestingly, the clinical activity of itraconazole was associated with down-modulation of Hh signaling in non-malignant tissue as measured by *GLI1* mRNA expression in serial skin biopsies. Importantly, itraconazole did not exert its activity via inhibition of adrenal androgen synthesis, which is the primary mechanism of action of a related azole antifungal, ketoconazole. More specifically, patients in the itraconazole study underwent serum measurements of both circulating testosterone and DHEA levels, both of which were not suppressed after treatment with itraconazole. However, it still remains possible that the clinical activity of high-dose itraconazole seen in this phase II study was not mediated by Hh pathway inhibition but possibly by other unknown off-target effects (potentially also including angiogenesis inhibition which has also been suggested from preclinical studies [32]).

Itraconazole has also demonstrated evidence of activity in the setting of biochemically-recurrent prostate cancer with non-castrate testosterone levels (*i.e.*, the so-called “rising PSA state” after definitive local therapy) [33]. This is evidenced from a case report of a hormone therapy-naive patient with biochemical recurrence following radical prostatectomy and salvage radiation therapy with PSA doubling time of 6 months. Due to the patient’s strong desire to avoid castrating therapies, off-label high-dose itraconazole treatment was initiated. After three months of therapy, he experienced a >50% PSA reduction, despite unchanged testosterone levels. Following 5 months of therapy with an ongoing PSA response, he developed asymptomatic hyperbilirubinemia, which necessitated discontinuation of itraconazole. Subsequent to drug discontinuation, his PSA began to rise once again. As mentioned above, while a related compound, ketoconazole, interrupts gonadal and adrenal steroidogenesis, itraconazole was not shown to suppress serum testosterone or DHEA levels in either the castrate or non-castrate settings. Finally, to formally test the clinical efficacy of itraconazole in men with non-castrate biochemically-recurrent prostate cancer following local therapy, a phase II trial using itraconazole in this setting has been launched (NCT01787331). The primary endpoint of this trial is the proportion of patients achieving a >50% decline in PSA while receiving itraconazole in the absence of other hormonal therapies (Table 1).

**Table 1:** Ongoing Clinical Trials of Hedgehog Inhibitors in Prostate Cancer.

National Clinical Trial Number	Target Population	Trial Name	Primary Endpoint	Status
NCT02111187	Localized disease/neoadjuvant	A Pre-surgical Study of Sonidegib in Men with High-risk Localized Prostate Cancer	Change from baseline in tissue GLI1 expression levels	Currently accruing
NCT01163084	Localized disease/neoadjuvant	Leuprolide Acetate or Goserelin Acetate With or Without Vismodegib Followed by Surgery in Treating Patients with Locally Advanced Prostate Cancer	Proportion of patients with less than 5% tumor involvement on prostatectomy	Active, not recruiting
NCT01787331	Rising PSA	A Phase II Study of Itraconazole in Biochemical Relapse	Proportion of patients who achieve greater than 50% decline in PSA	Currently accruing
NCT02115828	Castration-resistant prostate cancer	A Pharmacodynamic Study of Vismodegib in Men With Metastatic Castration-resistant Prostate Cancer (mCRPC) With Accessible Metastatic Lesions for Tumor Biopsy	Proportion of patients with at least a 50% reduction in tumor GLI1 expression level from baseline	Currently Accruing
NCT02182622	Castration-resistant prostate cancer	Sonidegib combined with Docetaxel for Met Castrate Resistant Prostate Cancer w/Disease Progression after Docetaxel	Maximum Tolerated Dose (MTD)	Not yet accruing

Given the potential clinical benefits seen with high-dose itraconazole in the CRPC setting that were associated with Hedgehog pathway inhibition in skin biopsies, and the relatively mild toxicity profiles in early-phase trials of more potent SMO inhibitors (intolerable grade-2 adverse events including dysgeusia, muscle spasms, and alopecia are notable), there has been increased interest in evaluating the clinical utility of these inhibitors in men with prostate cancer. To this end, two pharmacodynamic trials (one in the neoadjuvant setting prior to radical prostatectomy (NCT02111187), and one in men with metastatic CRPC (NCT02115828)) are currently accruing patients to determine the effect of potent small-molecule SMO inhibitors (sonidegib and vismodegib, respectively) on Hh pathway activity in the tumor and microenvironment directly, rather than using the skin as a surrogate tissue. In the first study (NCT02111187), patients with high-risk localized prostate cancer (based on biopsy Gleason grade, clinical stage, and PSA levels) who are considered candidates for radical prostatectomy are treated with 4 weeks of sonidegib or are observed for 4 weeks before undergoing definitive surgery. The primary endpoint of this neoadjuvant trial is a 50% decrease from baseline in prostatic tissue *GLI1* mRNA expression levels using quantitative reverse transcription PCR, while secondary endpoints include the rate of complete pathologic response, change in markers of apoptosis and proliferation, and risk of biochemical recurrence following prostatectomy. The investigators hypothesized that over 50% of patients treated with sonidegib will demonstrate a >50% reduction in *GLI1* mRNA expression, compared with fewer than 10% of patients treated with observation alone.

The other pharmacodynamic trial, NCT02115828, is exploring the effect of vismodegib in men with CRPC treated in the post-abiraterone or post-enzalutamide era who harbor at least one soft-tissue metastatic site that is amenable to a needle biopsy. Subjects will receive 4 weeks of vismodegib therapy and will undergo baseline and post-treatment metastatic tumor biopsies. As in the prior trial, the primary endpoint of this study is the proportion of subjects with a >50% decrease in *GLI1* mRNA expression levels in the post-treatment biopsies compared to the pre-treatment biopsies. Secondary endpoints in this study include evidence of clinical activity (progression-free survival, PSA response, overall survival) as well as correlative markers, including change in expression of other components of the Hh pathway (PTCH1, GLI2), and correlation of change in *GLI1* mRNA expression between skin and tumor biopsies. Technical hurdles with regards to GLI1 immunohistochemistry have hindered insights into the importance of epithelial *versus* stromal Hedgehog pathway activity. In this trial, tissue mRNA analysis of pre-treatment and post-treatment *GLI1* expression via RNA *in situ* hybridization (using RNAScope technology) is planned to better evaluate the dynamics of the Hedgehog pathway in the tumor microenvironment.

Next, a randomized phase I/II trial is targeting men with high-risk locally-advanced prostate cancer who are being randomized either to a GnRH agonist alone or combined with vismodegib in the neoadjuvant setting (NCT01163084). Patients in this trial will be treated according to their randomization arm for a period of 4 months prior to radical prostatectomy. The primary endpoint of this trial is the proportion of patients with pathologic response in their radical prostatectomy specimens (complete response or near-complete response: less than 5% tumor involvement). Secondary endpoints include biochemical relapse rate, rate of positive surgical margins, time to clinical/radiographic progression, and changes in Hh and androgen-signaling markers in prostatic tissue.

Finally, building on the data supporting reversal of chemo-resistance with Hedgehog inhibition, a phase Ib trial investigating the safety and tolerability of combining sonidegib with docetaxel in men with docetaxel-resistant CRPC is planned (NCT02182622). In this trial, patients who are experiencing clinical or radiographic progression of their disease despite standard chemotherapy treatment with docetaxel 75 mg/m^2^ given intravenously once every 3 weeks (as well as previous exposure to abiraterone and/or enzalutamide) will have oral sonidegib added to their docetaxel regimen using a classical dose-escalation design (level 1: sonidegib 200 mg daily; level 2: sonidegib 400 mg daily; level 3: sonidegib 800 mg daily). The primary endpoint of this study is the maximum tolerated dose (MTD) of the combination of sonidegib and docetaxel. If the combination is well-tolerated, this will likely lead to a randomized phase II trial comparing docetaxel alone to docetaxel plus sonidegib.

## 4. Future Directions

Investigations into the clinical utility of Hh pathway manipulation in prostate cancer remain in the early stages. Several important preclinical and clinical questions remain unanswered. What is the exact nature of the interaction between the Hh pathway and the androgen/AR pathways? Does increased Hh activity in the tumor microenvironment occur primarily via autocrine or paracrine pathways and, if it is the latter, what are the critical mediators between the stroma and tumor? Is SMO the optimal target to abrogate Hh signaling leading to the pleiotropic effects promoting prostate cancer growth? Will single-agent inhibition be sufficient in a particular clinical setting or are combinations with other standard therapies needed? Would Hh inhibition prove most useful in early or more advanced prostate cancer; in hormone-sensitive or castration-resistant disease? And, if Hh pathway inhibitors prove effective in prostate cancer, what will be the best strategy to treat tumor resistance? Experience in other histologies, particularly basal cell carcinoma, suggests the rapid development of resistance to single-agent SMO inhibitors [34]. With regards to this last question, Kim *et al.* demonstrated promising activity of the combination of arsenic trioxide (which promotes degradation of GLI2 (Figure 2)) and itraconazole in a vismodegib-resistant medulloblastoma mouse model [35]. Bromodomain extra-terminal (BET) inhibitors have also demonstrated intriguing preclinical activity in SMO-mutated tumors by disrupting the interaction of GLI1 and GLI2 with their respective promotor/enhancer sequences on DNA [36]. Another promising downstream inhibitor of Hh is glabrescione B, which binds the GLI1 zinc finger and impairs its interaction with DNA [37]. These strategies certainly warrant further investigation in the prostate cancer arena.

In addition, given that the use of Hh inhibitors as monotherapy in the clinical setting to date has been effective in basal cell carcinoma and medulloblastoma, but not in other tumor types, combination strategies will likely be needed in prostate cancer. In order to allow for the development and testing of rational combinations of Hh inhibitors with other agents, a better understanding of the interactions between the Hh pathway and other signaling pathways within prostate cancer cells is needed. Several strategies appear promising. For example, activation of Hedgehog signaling in hepatocellular carcinoma protected tumor cells against ionizing radiation, while Hh inhibition can radiosensitize tumors including non-small cell lung cancer and pancreatic cancer [38,39,40]. This potential synergy has not yet been evaluated in prostate cancer. Another pathway of interest is the PI3K-mTOR signaling pathway, which is altered in approximately half of primary prostate tumors and nearly all cases of metastatic castration-resistant disease [41]. It is now appreciated that there is crosstalk between these pathways, and upregulation of PI3K signaling may be a resistance mechanism for SMO inhibitors [42]. Concomitant blockade of the epidermal growth factor pathway and the Hh pathway appears synergistic in further sensitizing CRPC cells to docetaxel, potentially through induction of the sphingolipid, ceramide [43]. The combination of cyclopamine and gefitinib was associated with activity in the absence of chemotherapy as well [44]. Thus, combinations of Hh antagonists with inhibitors of the PI3K pathway may prove interesting in prostate cancer. Currently, several early-phase clinical trials of Hh pathway antagonists are ongoing; further investigation of Hh inhibitors both as monotherapies or combined with other standard therapies is warranted in patients with prostate cancer.
